# Sociodemographic inequities in nurturing care for early childhood development across Brazilian municipalities

**DOI:** 10.1111/mcn.13232

**Published:** 2021-07-06

**Authors:** Gabriela Buccini, Stefanie Eugênia dos Anjos Coelho Kubo, Jéssica Pedroso, Juracy Bertoldo, Alberto Sironi, Marcos Ennes Barreto, Rafael Pérez‐Escamilla, Sonia Isoyama Venancio, Muriel Bauermann Gubert

**Affiliations:** ^1^ Department of Environmental Health, School of Public Health University of Nevada Las Vegas Nevada USA; ^2^ Departamento de Nutrição Universidade de Brasilia Brasília Brazil; ^3^ Department of Computing Universidade Federal da Bahia Salvador Brazil; ^4^ Department of Social and Behavioral Sciences Yale School of Public Health New Haven Connecticut USA; ^5^ Núcleo de Evidências Instituto de Saúde da Secretaria de Estado da Saúde de São Paulo São Paulo Brazil

**Keywords:** Brazil, child development, cities, environmental indicator, index, monitoring, nurturing care, public health surveillance

## Abstract

Providing an enabling nurturing care environment for early childhood development (ECD) that cuts across the five domains of the Nurturing Care Framework (i.e., good health, adequate nutrition, opportunities for early learning, security and safety and responsive caregiving) has become a global priority. Brazil is home to approximately 18.5 million children under 5 years of age, of which 13% are at risk of poor development due to socio‐economic inequalities. We explored whether the Early Childhood Friendly Municipal Index (IMAPI) can detect inequities in nurturing care ECD environments across the 5570 Brazilian municipalities. We examined the validity of the IMAPI scores and conducted descriptive analyses for assessing sociodemographic inequities by nurturing care domains and between and within regions. The strong correlations between school achievement (positive) and socially vulnerable children (negative) confirmed the IMAPI as a multidimensional nurturing care indicator. Low IMAPI scores were more frequent in the North (72.7%) and Northeast (63.3%) regions and in small (47.7%) and medium (43.3%) size municipalities. Conversely, high IMAPI scores were more frequent in the more prosperous South (52.9%) and Southeast (41.2%) regions and in metropolitan areas (41.2%). The security and safety domain had the lowest mean differences (MDs) among Brazilian regions (MD = 5) and population size (MD = 3). Between‐region analyses confirmed inequities between the North/Northeast and South/Southeast. The biggest within‐region inequity gaps were found in the Northeast (from −22 to 15) and the North (−21 to 19). The IMAPI distinguished the nurturing care ECD environments across Brazilian municipalities and can inform equitable and intersectoral multilevel decision making.

**Key messages**

- The Brazilian Early Childhood Friendly Municipal Index (IMAPI) measures the nurturing care dimensions of the early childhood development (ECD) environment (i.e., good health, adequate nutrition, opportunities for early learning, security and safety and responsive caregiving).
- The IMAPI provides a robust summary measure of the enabling nurturing care environment for ECD and can facilitate the development of equitable and intersectoral integrated ECD policies and programmes from the national to the municipal level.
- The IMAPI documented great sociodemographic inequities in the enabling nurturing care environments for ECD across Brazilian municipalities.
- The experience of the IMAPI may be beneficial to other countries also experiencing strong social and ECD inequities.

## 1 INTRODUCTION

Providing a nurturing care environment for early childhood development (ECD) that cuts across the five domains of the Nurturing Care Framework (i.e., good health, adequate nutrition, opportunities for early learning, security and safety and responsive caregiving) has become a global priority to ensure that children survive and thrive (Black et al., 2017, 2021; Britto et al., 2017; Richter et al., 2017; World Health Organization [WHO], 2019). Brazil is the largest country in South America, with approximately 18.5 million children under 5 years of age (9% of the Brazilian population) (Instituto Brasileiro de Geografia e Estatistica [IBGE], 2017). Their optimal development is critical to the human capital development of the country (Black et al., 2017, 2021; Lu et al., 2020; Shonkoff et al., 2012). However, Brazil lacks a systematic monitoring system with disaggregated ECD data. In 2015, an estimated 13% of children under the age of 5 were at risk of poor development due to stunting or extreme poverty (Richter et al., 2017). Brazil is a very inequitable society, which is captured through the great socio‐economic variation across regions and the 5570 municipalities (Instituto de Pesquisa Econômica Aplicada [IPEA], 2015). These inequities are also highly visible as a function of skin colour, with Black and Brown individuals being much more likely to live in poverty irrespective of which region in the country that they live. They are 56% of the Brazilian population and represent 73% of the poor in Brazil (IBGE, 2017).

Brazil experiences strong inequities in child poverty and maternal health and childcare (Aristides dos Santos et al., 2019). Hence, strengthening evidence‐based ECD policies and programmes for intersectoral nurturing care should be a top priority in the country. This is especially important for the future of the nation as nurturing care has been identified as critical to reduce inequities and as the foundation for equitable human and social development (Black et al., 2017, 2021; Morris et al., 2017) and for countries to meet the Sustainable Development Goals (Black et al., 2017, 2021; Britto et al., 2017; Richter et al., 2017).

The Brazilian 2016 Legal Framework for ECD (Câmara dos Deputados, 2016), which aligns well with the WHO/UNICEF/World Bank Nurturing Care Framework (Black et al., 2017, 2021; Britto et al., 2017; Richter et al., 2017; WHO, UNICEF, & World Bank, 2018), outlines the importance of strengthening ECD systems to break the cycle of poverty by ensuring that all children, especially the most vulnerable, reach their development potential over their life course. Therefore, in recent years, the ECD agenda in Brazil has been strengthened, which has led to strong increases in investments and efforts to implement ECD programmes, targeting the most socio‐economically vulnerable children in the country, on a large scale. The majority of these investments have gone into the Criança Feliz (‘Happy Child’ programme), a national home visiting programme that has already been implemented in about 3000 Brazilian municipalities. Unfortunately, the programme has run into many obstacles (Buccini, Pedroso et al., 2021), because Brazil, as many other countries, still lacks a systematic approach to collect data that support evidence‐informed equitable and intersectoral decision making to improve integrated ECD policies and programmes based on the Nurturing Care Framework.

The Nurturing Care Framework includes a global call to monitor ECD environments across nurturing care domains (Richter et al., 2019, 2020; WHO, 2019), especially in the context of highly inequitable societies like Brazil. In response to this call, we developed the Early Childhood Friendly Municipal Index (IMAPI—*Índice Município Amigo da Primeira Infância*), which as far as we know is the first attempt to assess nurturing care for ECD at the municipal level (Buccini et al., 2021). The IMAPI is computed with a large amount of municipal‐level data derived from public databases in the critical ECD areas of health, education and social development. The data are integrated into a single score to monitor the overall nurturing care environment for ECD. The aims of this study were to explore the potential of the IMAPI to assess the nurturing care environments at the municipal level and elucidate whether it can detect sociodemographic inequities in the nurturing care ECD environment(s) across the 5570 Brazilian municipalities.

## 2 METHODS

### 2.1 Study settings

This is an ecological study designed to generate and analyse IMAPI scores in the 5570 Brazilian municipalities within 26 states and the Federal District. The IMAPI was developed following an eight‐steps methodology (Appendix S1). The first three steps involved a participatory multisectoral decision‐making process to identify nurturing care indicators (Buccini et al., 2021). A complete list of selected nurturing care indicators can be found in Appendix S2. Steps 4 to 6 consisted of statistical methods used to analyse and standardize nurturing care indicators: (i) data were requested and extracted from Brazilian government databases; (ii) consistency analysis was conducted individually for each indicator, and missing data were imputed; and (iii) indicators were standardized. Finally, in Steps 7 and 8, the set of 31 indicators available at the municipal level in the Brazilian databases between 2015 and 2019 was summarized into five subscores representing the Nurturing Care Framework domains: good health (*n* = 14), adequate nutrition (*n* = 4), opportunities for early learning (*n* = 7), security and safety (*n* = 5) and responsive caregiving (*n* = 1). Following the statistical criteria of having at least two indicators in the subscore domain to be included in the overall IMAPI score, the responsive caregiving domain was excluded. The overall IMAPI score is composed of 30 indicators across four Nurturing Care domains. The overall IMAPI score and subscores ranged from 0 to 100, and scores were categorized in high, medium and low categories based on the corresponding tercile distributions. The detailed description of methods to impute, standardize and estimate IMAPI scores can be found in Appendix S3.

### 2.2 Data analysis

**Figure 1:**
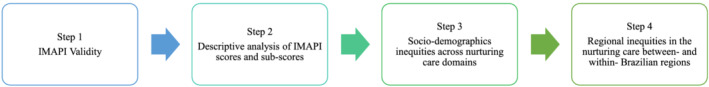
Analytical steps to assess sociodemographic inequities in the nurturing care environments

Four analytical steps were followed to assess sociodemographic inequities in the nurturing care environments (Figure 1). Statistical analyses were conducted in Stata 14.2 and SPSS 21.0.

#### 2.2.1 Step 1. Validity of the IMAPI scores

The validation process was conducted to find out if the IMAPI could provide a metric that captures the multiple dimensions of the nurturing care environment (purpose) in Brazilian municipalities (context) (Frongillo, 1999). The predictive validity (i.e., how well one measure predicts an outcome or measure) and the concurrent validity (i.e., how well one measure estimates a related condition present at approximately the same time) (Lin & Yao, 2014) were the two validity approaches used to test whether the IMAPI captured the different dimensions of the nurturing care environment for ECD across municipalities. Because data about ECD outcomes in Brazil are currently unavailable, the outcome considered in the predictive validity analysis was the 2017 Basic Education Development Index (IDEB). Based on a multidimensional scale, the IDEB summarizes elementary‐aged children's school achievement (i.e., enrolment, proficiency and success), and IDEB scores range from 0 to 10 (Chirinea & Brandao, 2015; Organization for Economic Co‐operation and Development [OECD], 2015). We hypothesized that an enabling nurturing care environment would be associated with a higher proportion of children with optimal development and school readiness. Previous studies have indicated that higher IDEB scores reflect the maximum benefit from both pre‐school and formal education (OECD, 2015; WHO, UNICEF, & World Bank, 2018). In the concurrent validity analysis, the outcome considered was the number of vulnerable children, that is, children under the age of 5 living in socio‐economic vulnerable families (i.e., monthly income of up to half a minimum wage per person or total monthly family income of up to three minimum wages) registered in the National Social Assistance Registry (CADÚNICO) (IBGE, 2017). We hypothesized that an enabling nurturing care environment would be associated with a lower proportion of at‐risk vulnerable children, as suggested in previous studies (Lu et al., 2016; Richter et al., 2017; Walker et al., 2011). Validity approaches were assessed through Spearman correlations between IMAPI scores and predictive (IDEB) and concurrent (vulnerable children) outcomes. Positive or negative correlations were classified as negligible (0.00–0.19), weak (0.20–0.29), moderate (0.30–0.39), strong (0.40–0.69) or very strong (0.70–1.00) relationships (Akoglu, 2018; Schober et al., 2018). A *p* value < 0.05 was the criterion for statistical significance.

#### 2.2.2 Step 2. Descriptive analysis of IMAPI scores and subscores

The overall IMAPI‐municipality score and subscores of the 5570 Brazilian municipalities are illustrated in maps. IMAPI scores were colour coded in three categories high (green), medium (yellow) and low (red) based on the corresponding tercile distributions.

#### 2.2.3 Step 3. Sociodemographic inequities across nurturing care domains

First, overall IMAPI score and subscores were described across three sociodemographic indicators: (1) Brazilian region (IBGE, 2017): North, Northeast, Central‐West, Southeast and South; (2) municipality population size (IBGE, 2017): very small (up to 20,000 inhabitants), small (20,001 to 50,000), medium (50,001 to 100,000), large (100,001 to 900,000) and metropolis (>900,000); and (3) proportion of vulnerable children, that is, children under the age of 5 from socially vulnerable families registered in the National Social Assistance Registry (CADÚNICO) (IBGE, 2017). The proportion of vulnerable children was analysed as either as continuous based on mean values or classified into categories—very low (up to 20%), low (20% to 30%), medium (30% to 40%), high (40% to 50%) and very high (over 50%)—according to the Social Vulnerability Index (IPEA, 2015). The description of municipalities according to the three‐selected demographic indicators is provided in Appendix S4. Differences in scores across categories were explored using the chi‐square test, and a *p* value < 0.05 was the criterion for statistical significance. Then, we estimated the mean IMAPI score and subscores by sociodemographic indicators and calculated the mean difference (MD) between the highest and the lowest mean scores.

#### 2.2.4 Step 4. Regional inequities between and within Brazilian regions

For the between‐group analyses, we classified state‐level IMAPI scores (which correspond to the mean scores of all municipalities within that state) as high, medium and low according to their tercile distributions. The difference between overall state IMAPI and the national IMAPI scores were calculated. For the within‐group analysis, we selected the five municipalities with the highest and the five with the lowest overall IMAPI scores in each region. The difference between the overall municipal and national IMAPI scores was used to estimate the size of the differences within regions.

## 3 RESULTS

**Figure 2:**
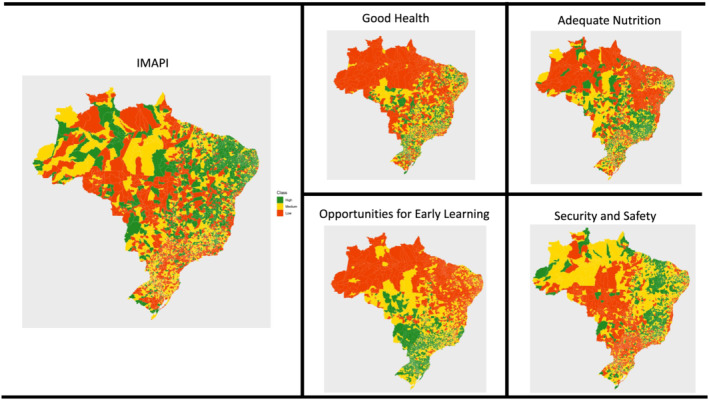
Spatial distribution of overall IMAPI and domain subscores in the 5570 Brazilian municipalities

The overall IMAPI score was strongly correlated with the IDEB (*r* = 0.61, *p* < 0.01) and vulnerable children (*r* = −0.48, *p* < 0.01), which confirms its potential to capture the multiple dimensions of an enabling nurturing care environment for ECD across municipalities. The overall IMAPI score and subscores across municipalities are presented in Figure 2. The overall IMAPI scores for 2170 (39.0%) municipalities were low, 1658 (29.8%) were medium and 1742 (31.3%) were high. Around a third of municipalities exhibited low IMAPI subscores (Appendix S3).

The IMAPI scores and subscores were able to document great sociodemographic inequities in the enabling nurturing care ECD environments across Brazilian municipalities. Low overall IMAPI scores were more frequent in municipalities in the North (72.7%) and Northeast (63.3%) regions of the country and in municipalities with small (47.7%) and medium (42.3%) population size. By contrast, high overall IMAPI scores were more frequent in municipalities in the South (52.9%) and in municipalities with over 900,000 inhabitants (41.2%) (Table 1). Whereas *low subscores* in good health, adequate nutrition and opportunities for early learning were more frequent in municipalities in the North and Northeast, *low subscores* in security and safety were more frequent in the Central‐West, closely followed by the South. *High subscores* in good health (39.9%) and security and safety (31.1%) were more frequent for very small population size municipalities; by contrast, *high subscores* in adequate nutrition (94.1%) and opportunities for early learning (52.9%) were more frequent in metropolitan areas. The IMAPI scores were also found to be associated with the proportion of vulnerable children (Table 1). The MDs in IMAPI scores across Brazilian regions, municipal population size and proportion of vulnerable children confirmed strong sociodemographic inequities in the strength of nurturing care ECD environments. The security and safety domain had the lowest MDs according to Brazilian regions (MD = 5) and municipal population size (MD = 3). By contrast, opportunities for early learning had the highest MDs across Brazilian regions (MD = 23) and proportion of vulnerable children (MD = 19) but not for municipal population size (Table 2).

**Table 1:** Sociodemographic characteristics of the municipalities in relation to the four domains included in the IMAPI

Sociodemographic characteristics of the municipalities		Total (*n*)	IMAPI^a^			Good health^a^			Adequate nutrition^a^			Opportunities for early learning^a^			Security and safety^a^		
			High (%)	Medium (%)	Low (%)	High (%)	Medium (%)	Low (%)	High (%)	Medium (%)	Low (%)	High (%)	Medium (%)	Low (%)	High (%)	Medium (%)	Low (%)
Regions	North	450	6.0	21.3	72.7	8.7	20.4	70.9	20.0	25.1	54.9	4.0	25.6	70.4	19.8	48.9	31.1
	Northeast	1784	5.7	31.0	63.3	22.7	34.1	43.2	16.1	24.4	59.5	1.7	29.1	69.2	37.8	46.4	15.8
	Southeast	1668	41.2	43.7	15.0	36.6	36.9	26.6	51.1	26.3	22.7	39.6	46.8	13.7	31.1	14.8	44.1
	South	1191	52.9	40.1	7.1	50.4	29.2	20.3	35.7	39.9	24.4	75.8	23.2	1.0	13.9	30.2	55.8
	Central‐West	467	22.5	50.5	27.0	31.7	35.1	33.2	27.2	41.8	31.9	37.7	46.7	15.6	6.1	30.8	63.0
Population size	Very small	3811	32.5	37.4	30.1	39.9	30.0	30.1	35.2	31.5	33.4	32.6	34.0	33.4	31.1	34.6	34.2
	Small	1100	15.9	36.7	47.7	19.5	38.8	41.6	20.8	28.6	50.5	25.9	31.7	42.4	19.7	40.6	39.6
	Medium	350	16.3	41.4	42.3	12.9	40.9	46.3	21.7	27.7	50.6	36.3	34.9	28.9	13.4	36.6	50.0
	Large	292	25.7	38.7	35.6	8.2	38.4	53.4	41.4	16.1	42.5	42.1	46.2	11.6	8.6	25.0	66.4
	Metropolis	17	41.2	47.1	11.8	0.0	23.5	76.5	94.1	0.0	5.9	52.9	47.1	0.0	5.9	23.5	70.6
Proportion of vulnerable children (mean)		‐	41.8	48.1	61.6	47.5	49.8	56.8	49.8	50.0	54.1	31.5	50.2	67.0	62.7	56.6	37.1

^a^ *p* < 0.001 for all characteristics.

**Table 2:** Means differences between highest and lowest IMAPI scores and subscores across regions and population size

Municipalities characteristics		IMAPI	Good health	Adequate nutrition	Opportunities for early learning	Security and safety
Region	North	38	48	23	57	22
	Northeast	40	56	22	58	24
	Southeast	47	60	31	72	23
	South	48	63	28	80	21
	Central‐West	44	58	27	72	19
	Mean differences	10	15	9	23	5
Population size	Very small	45	60	27	68	23
	Small	42	55	23	66	22
	Medium	44	54	25	69	21
	Large	42	53	31	73	20
	Metropolis	47	49	44	74	20
	Mean differences	5	11	21	8	3
Proportion of vulnerable children	Very low	47	63	27	80	19
	Low	46	60	28	77	20
	Medium	45	59	28	73	21
	High	44	58	27	70	22
	Very high	41	56	24	61	25
	Mean differences	6	7	4	19	6

When comparing the difference, as a function of sociodemographic characteristics, between and within each region, we found marked differences in the strength of the enabling nurturing care ECD environment. Between‐region analyses confirmed greater negative differences between state IMAPI scores and national IMAPI scores in the North and Northeast regions. By contrast, greater positive differences were found in most of the states in the South (Table 3). Only *Rio Grande do Norte* (located in the Northeast) and *Minas Gerais* (located in the Southeast) were in the high IMAPI category for security and safety; half of the states in the Southeast and Central‐West were in the low IMAPI category for security and safety (Table 3). Within regions, inequities were marked by the largest negative difference between the overall municipal and the national IMAPI scores within the Northeast (from −22 to 15) and the North (−21 to 19) regions, respectively (Table 4).

**Table 3:** Difference between the national and state IMAPI scores and subscores across Brazilian regions

Region	States	IMAPI‐state score and subscores									
		IMAPI	Diff state‐national scores	Good health	Diff state‐national scores	Adequate nutrition	Diff state‐national scores	Opportunities for early learning	Diff state‐national scores	Security and safety	Diff state‐national scores
North	Tocantins	43	−1	55	−3	31	5	62	−6	24	2
	Rondônia	41	−3	57	−1	26	0	64	−4	18	−4
	Roraima	37	−7	43	−15	23	−3	57	−11	24	2
	Acre	35	−9	46	−12	15	−11	52	−16	25	3
	Amazonas	34	−10	44	−14	15	−11	52	−16	24	2
	Pará	34	−10	41	−17	19	−7	54	−14	21	−1
	Amapá	34	−10	41	−17	18	−8	53	−15	24	2
Northeast	Paraíba	44	0	63	5	29	3	59	−9	25	3
	Ceará	42	−2	60	2	22	−4	63	−5	23	1
	Pernambuco	41	−3	58	0	27	1	57	−11	23	1
	Rio Grande do Norte	41	−3	56	−2	23	−3	62	−6	22	0
	Piauí	40	−4	54	−4	21	−5	57	−11	28	6
	Sergipe	40	−4	56	−2	25	−1	58	−10	23	1
	Alagoas	40	−4	54	−4	25	−1	56	−12	24	2
	Bahia	38	−6	53	−5	19	−7	58	−10	23	1
	Maranhão	34	−10	49	−9	12	−14	50	−18	25	3
Southeast	Minas Gerais	49	5	62	4	36	10	70	2	27	5
	Espirito Santo	48	4	60	2	30	4	79	11	22	0
	São Paulo	45	1	59	1	26	0	76	8	18	−4
	Rio de Janeiro	42	−2	52	−6	25	−1	70	2	19	−3
South	Santa Catarina	49	5	64	6	27	1	83	15	21	−1
	Rio Grande do Sul	48	4	62	4	26	0	80	12	22	0
	Paraná	47	3	63	5	29	3	79	11	18	−4
Central‐West	Distrito Federal	55	11	60	2	58	32	79	11	22	0
	Mato Grosso do Sul	46	2	54	−4	27	1	81	13	21	−1
	Goiás	44	0	59	1	26	0	70	2	20	−2
	Mato Grosso	44	0	58	0	27	1	71	3	18	−4

*Note:* IMAPI‐state score and subscores correspond to the mean performances of all municipalities within that state. Categories were classified as high (green color), medium (yellow color) and low (red color) according to tercile.

**Table 4:** Municipalities with highest and lowest IMAPI scores within Brazilian regions

Region	Municipalities	State	Population size	IMAPI‐municipal score	Diff municipal‐national IMAPI score^a^
North (*n* = 450)	Municipalities with HIGHEST IMAPI scores				
	Presidente Kennedy	TO	Very small	63	19
	Brasilândia do Tocantins	TO	Very small	62	18
	Araguaíana	TO	Large	57	13
	Jaú do Tocantins	TO	Very small	55	11
	Tupirama	TO	Very small	55	11
	Municipalities with LOWEST IMAPI scores				
	Barcelos	AM	Small	23	−21
	Igarapé‐Miri	PA	Medium	24	−20
	Feijó	AC	Small	25	−19
	Chaves	PA	Small	25	−19
	Jordão	AC	Very small	25	−19
Northeast (*n* = 1794)	Municipalities with HIGHEST IMAPI scores				
	Serra Negra do Norte	RN	Very small	59	15
	Farias Brito	CE	Very small	56	13
	Areia de Baraúnas	PB	Very small	56	13
	São Bentinho	PB	Very small	55	11
	Sebastião Leal	PI	Very small	55	11
	Municipalities with LOWEST IMAPI scores				
	Fernando Falcão	MA	Very small	22	−22
	Paquetá	PI	Very small	22	−22
	Tupanatinga	PE	Small	22	−22
	Presidente Juscelino	MA	Very small	25	−19
	Lajedão	BA	Very small	25	−19
Southeast (*n* = 1668)	Municipalities with HIGHEST IMAPI scores				
	Umburatiba	MG	Very small	70	26
	São Sebastião do Anta	MG	Very small	67	23
	Monjolos	MG	Very small	67	23
	Senador Modestino Gonçalves	MG	Very small	67	23
	Cedro do Abaeté	MG	Very small	67	23
	Municipalities with LOWEST IMAPI scores				
	Itambé do Mato Dentro	MG	Very mall	23	−21
	Queimados	RJ	Large	24	−20
	Belford Roxo	RJ	Large	25	−19
	Lagoa dos Patos	MG	Very small	25	−19
	Itaóca	SP	Very small	25	−19
South (*n* = 1191)	Municipalities with HIGHEST IMAPI scores				
	Coronel Barros	RS	Very small	74	30
	Novo Horizonte	SC	Very small	68	24
	Bela Vista da Caroba	PR	Very small	66	22
	Uruguaiana	RS	Large	65	21
	Cotiporã	RS	Very small	64	20
	Municipalities with LOWEST IMAPI scores				
	São José do Cerrito	SC	Very small	30	−14
	Canudos do Vale	RS	Very small	34	−10
	Santo Antônio do Palma	RS	Very small	35	−9
	Alvorada	RS	Large	35	−9
	Rancho Alegre D'Oeste	PR	Very small	36	−8
Central‐West (*n* = 467)	Municipalities with HIGHEST IMAPI scores				
	Jaupaci	GO	Very small	63	19
	Campo Grande	MS	Large	57	13
	Israelândia	GO	Metropolis	55	11
	Reserva do Cabaçal	MT	Very small	55	11
	Brasília	DF	Very small	55	11
	Municipalities with LOWEST IMAPI scores				
	Maurilândia	GO	Very small	27	−17
	Castelândia	GO	Very small	30	−14
	Tesouro	MT	Very small	30	−14
	Araguaiana	MT	Very small	32	−12
	Guapó	GO	Very small	33	−11

^a^ IMAPI‐national score is 44 and corresponds to the mean scores of all Brazilian municipalities.

## 4 DISCUSSION

The IMAPI experience in Brazil revealed the complexity of measuring a multidimensional construct such as the nurturing care environment for ECD. Our analyses showed that the IMAPI had enough resolution to distinguish municipalities according to the level of strength that each exhibited for nurturing care environments. We found strong sociodemographic inequities in nurturing care environments between and within Brazilian regions, municipality population size and proportion of vulnerable children. Hence, IMAPI scores and subscores can be used as a summary measure to differentiate settings according to the strength in their enabling nurturing care environments and have the potential to help inform the development of improved evidence‐based equitable and intersectoral multilevel decision making. The IMAPI can help advance monitoring and strengthening of nurturing care environments in other large countries also experiencing significant social and ECD inequities, such as Mexico, China and India.

The quantitative validity analyses of the IMAPI presented in this study, combined with the construct validity findings previously reported (Buccini et al., 2021), confirmed the ability of the IMAPI to capture the strength of the multiple dimensions of an enabling nurturing care ECD environment across Brazilian municipalities. The strong negative correlations between the overall IMAPI scores with proportion of vulnerable children and positive correlations with IDEB scores confirmed both of our hypotheses—an enabling nurturing care environment for ECD would be associated with a lower proportion of at‐risk children (Lu et al., 2016; Lu et al., 2020; Richter et al., 2019) and predict a maximum benefit for the child's formal education (OECD, 2015; WHO, UNICEF, & World Bank, 2018). Our findings illustrate the importance of interpreting the strength of these correlations within the context of the complexity of measuring a multidimensional and intersectoral construct such as the Nurturing Care Framework (Schober et al., 2018).

Among the nurturing care domains, the security and safety domain had the lowest MDs according to Brazilian regions and municipal population size. Our findings on ‘security’ (levels of child's family social protection) may reflect the reach of the social protection services, including the Brazilian conditional cash transfer programme for the most vulnerable families, which has been shown to be a potent intersectoral policy for reducing inequities (Neves et al., 2020; Palmeira et al., 2020). However, socio‐economic inequities in the ‘security’ domain may have been hidden by the lack of a robust process for notifying violence against women and children (Assis et al., 2012; Silva & Roncalli, 2020). Similarly, our findings on ‘safety’ (degree of community safety) confirmed recent analyses showing a decreased inequity gap between the number of homicides in metropolitan areas and in small and less urbanized municipalities (Nsoesie et al., 2020). On the other hand, the fact that air pollution affects mainly urban areas where 85% of the Brazilian population live may have influenced the relatively low MDs in the ‘safety’ domain when analysed by region and municipal population size. Collectively, these facts may explain, at least in part, the lower MDs in sociodemographic inequities in the security and safety domain.

Opportunities for early learning exhibited the highest MD between Brazilian regions and proportion of children in social vulnerability. Inequities in access to early education for children from 0 to 6 years across regions were pronounced—ranging from 33.9% to 49.2% in the North and Southeast regions, respectively—and confirmed by the contrasting levels of illiteracy (15 years of age or older) in the Northeast (20.0%) and South (4.5%) regions (Conselho de Desenvolvimento Econômico e Social [CDES], 2014; OECD, 2015). These findings are consistent with our previous analysis illustrating that academic success since early childhood is as a function of the social development of a region; hence, educational interventions need to focus on the most socio‐economically and geographically vulnerable populations (Arsenault et al., 2017; Black et al., 2021).

Regional social inequities are a well‐known problem in Brazil (Aristides dos Santos et al., 2019; Gubert et al., 2017), and our study confirmed the strong inequities in nurturing care environments, especially within the most impoverished regions of the country—the Northeast and North —where the largest inequity gaps in nurturing care environments were found. There was a very strong contrast between the low IMAPI scores in the North/Northeast compared with the high IMAPI scores in the South/Southeast. Despite the slow progress the country has made in reducing regional inequities over the past decades that have helped children survive (Sengupta, 2019; Silva & Paes, 2019), in‐depth structural inequities, such as income inequality (Aristides dos Santos et al., 2019; Reis, 2014), racial gaps in education and discrimination (Salata, 2020) and child poverty, are still denying vulnerable children access to a high‐quality nurturing care environment that would allow them to thrive (Black et al., 2021).

Moving beyond regional disparities, the IMAPI further advances the contributions from existing population‐based tools such as the Countdown to 2030 early childhood country profile (UNICEF, 2020) and the State of Babies in the United States (Keating et al., 2020), as it makes estimates at the municipal level. Hence, the IMAPI can identify inequities in nurturing care environments not only across but also within geographical areas. Therefore, our findings call for the need to address geographical and social exclusion (Arsenault et al., 2017) and support the expected central role of municipalities (Avellaneda, 2012), as established in the Brazilian Federative Pact, to build their own destinies following local decision‐making roadmaps to manage and set priorities to fight nurturing care inequities.

Our findings showed that low IMAPI scores were more frequent in small and medium population size than very small and large municipalities, perhaps reflecting different challenges for nurturing care environments as a function of municipal population size (Avellaneda & Gomes, 2015; Wehrmeister et al., 2017). On the one hand, very small population size municipalities may have less capacity and less financial independence to invest in diversifying the offer of ECD‐related programmes as they must rely more on federal‐funded programmes (Avellaneda & Gomes, 2015). On the other hand, they may have more control over the integration and governance of ECD‐related systems and programmes, which are critical aspects for enabling nurturing care (Britto et al., 2014) especially among the most socially vulnerable children (Wehrmeister et al., 2017). By contrast, a metropolis may have more challenges related to rapid urbanization and population growth leading to higher indices of violence and difficulty reaching the population living in the most impoverished peripheral neighbourhoods. This is illustrated in an in‐depth systematic analysis of the implementation of the home visiting Criança Feliz parenting skills programme (Buccini, Pedroso, et al., 2021). Criança Feliz has faced scaling up challenges in capitals and metropolises due to complex logistical challenges of urbanization, such as long distances between homes, difficulty scheduling visits, insufficient federal funding, limited existing infrastructure and poor internet access (Buccini, Pedroso, et al., 2021). Furthermore, in Brazil, the population size of the municipality is important as it determines the amounts of federal financial transfers and incentives to equalize the income across municipalities, which has been critical for municipalities with very small populations, especially given their fragile economic and social structure (Massardi & Abrantes, 2016). In this sense, very small population size municipalities seemed to benefit from this financial equalization, as in our analysis, they presented better scores, which translate into more equitable nurturing care environments than small and medium population size municipalities (Wehrmeister et al., 2017). These findings call for specific financial protection and implementation strategies for advancing equity in nurturing care in municipalities with small and medium populations (Arsenault et al., 2017; Wehrmeister et al., 2017), which correspond to about 30% of the Brazilian municipalities.

A major strength of the IMAPI is that it combines a high volume of information from indicators of different disciplines and produces a summary measure and submeasures of enabling environments that are needed for proper ECD. The IMAPI was carefully and systematically developed following a series of methodological steps to summarize indicators representing the enabling environment for nurturing care. However, we acknowledged several limitations of our study. First, responsive caregiving was limited to one indicator and therefore did not meet the statistical criteria to be included in the overall IMAPI scores (Buccini et al., 2021; Appendix S3). This limitation was not unexpected given the challenge of measuring responsive caregiving globally (UNICEF, 2020). Second, as expected, the security and safety domain were challenging to interpret due to the bidirectional dimension of this domain (WHO, 2019), which sometimes can lead to counterintuitive findings. In the case of the IMAPI, ‘security’ measured levels of children's social protection (i.e., coverage of the conditional cash transfer and notification of violence against women and children), and ‘safety’ measured the degree of community safety or lack of it (i.e., homicides and air pollution). For instance, the authors had a substantive debate about how to interpret the ‘notification of violence against children and women’ indicators. On the one hand, these indicators could be interpreted as being protective for ECD—more notifications of violence bring more supportive services to a community. On the other hand, they could be indicators of risk for ECD—higher number of violence notifications could simply reflect higher level of violence in the community. This is because, although the notification of violence against children and women is mandatory in Brazil, it is not a formal charge, but rather an instrument to guarantee human rights; thus, in this sense, it should be interpreted as a protective community factor and, consequently, inversely related to inequalities in child health as suggested in previous studies (Wehrmeister et al., 2017). Third, the adequate nutrition domain was composed of indicators from the Brazilian Food and Nutrition Surveillance System (SISVAN), which collects continuous information on the nutritional status and food consumption of children and adolescents receiving primary health care services (Mourão et al., 2019). We originally planned to select individual‐level indicators (e.g., breastfeeding and prevalence of overweight/obesity) to compose IMAPI scores. However, due to the low coverage of individual‐level nutritional indicators in the SISVAN across municipalities (Mourão et al., 2019), the IMAPI evaluated the nutritional surveillance capacity of the municipalities through municipal‐level aggregated information (the proportion of children with information in the SISVAN). Finally, we acknowledge that the IMAPI should be refined as more indicators across nurturing care domains become available, including counselling programmes addressing responsive feeding (Pérez‐Escamilla et al., 2021).

In summary, the IMAPI, which is rooted in the Nurturing Care Framework, is a simple and useful population‐based tool to summarize the strength of and identify inequities in nurturing care at the regional and municipal levels. Hence, the IMAPI can help guide a more accurate and in‐depth understanding of where the major gaps are in the nurturing care environments across municipalities, ultimately favouring equitable policies and smart investments.

## CONFLICTS OF INTEREST

The authors have none conflicts to declare.

## CONTRIBUTIONS

GB, MEB and MBG conceptualized the Brazilian Early Childhood Friendly Municipal Index (IMAPI). GB and SEACK analysed the data with the support of JP, AS and JB and intellectual input of MBG. GB interpreted the data and wrote the first draft of the manuscript, and MBG, MEB, RPE and SIV revised it critically through an iterative process. All authors revised and approved the final version of the manuscript.

## Supporting information


**Appendix S1.**. Eight‐step systematic methodology to develop the Brazilian Early Childhood Friendly Municipal Index (IMAPI).

**Appendix S2.**. Standard definitions of the selected indicators composing the Brazilian Early Childhood Friendly Municipal Index (IMAPI).

**Appendix S3.**. Technical note on methods used to impute, standardize, and calculate IMAPI scores

**Appendix S4.**. Demographic characteristics of the 5.570 Brazilian municipalities included in the IMAPI.

## Data Availability

The data that support the findings of this study are available from the corresponding author upon reasonable request.

## References

[mcn13232-bib-0001] Akoglu, H. (2018). User's guide to correlation coefficients. Turkish Journal of Emergency Medicine, 18(3), 91–93. 10.1016/j.tjem.2018.08.001 30191186 PMC6107969

[mcn13232-bib-0002] Aristides dos Santos, A. M. , Perelman, J. , Jacinto, P. A. , Tejada, C. , Barros, A. , Bertoldi, A. D. , Matijasevich, A. , & Santos, I. S. (2019). Income‐related inequality and inequity in children's health care: A longitudinal analysis using data from Brazil. Social Science & Medicine (1982), 224, 127–137. 10.1016/j.socscimed.2019.01.040 30772611 PMC6411923

[mcn13232-bib-0003] Arsenault, C. , Johri, M. , Nandi, A. , Rodríguez, J. M. M. , Hansen, P. M. , & Harper, S. (2017). Country‐level predictors of vaccination coverage and inequalities in Gavi‐supported countries. Vaccine, 35(18), 2479–2488. 10.1016/j.vaccine.2017.03.029 28365251

[mcn13232-bib-0004] Assis, S. G. , Avanci, J. Q. , Pesce, R. P. , Pires, T. O. , & Gomes, D. L. (2012). Notificações de violência doméstica, sexual e outras violências contra crianças no Brasil. Ciência & Saúde Coletiva, 17(9), 2305–2317. 10.1590/S1413-81232012000900012 22996882

[mcn13232-bib-0005] Avellaneda, C. N. (2012). Mayoral decision‐making: Issue salience, decision context, and choice constraint? An experimental study with 120 Latin American mayors. Journal of Public Administration Research and Theory, 23, 631–661.

[mcn13232-bib-0006] Avellaneda, C. N. , & Gomes, R. C. (2015). Is small beautiful? Testing the direct and nonlinear effects of size on municipal performance. Public Administration Review, 75, 137–149. 10.1111/puar.12307

[mcn13232-bib-0007] Black, M. M. , Behrman, J. R. , Daelmans, B. , Prado, E. L. , Richter, L. , Tomlinson, M. , Trude, A. C. B. , Wertlieb, D. , Wuermli, A. J. , & Yoshikawa, H. (2021). The principles of Nurturing Care promote human capital and mitigate adversities from preconception through adolescence. BMJ Global Health, 6, e004436. 10.1136/bmjgh-2020-004436 PMC8057542 33875519

[mcn13232-bib-0008] Black, M. M. , Walker, S. P. , Fernald, L. C. H. , Andersen, C. T. , DiGirolamo, A. M. , Lu, C. , McCoy, D. C. , Fink, G. , Shawar, Y. R. , Shiffman, J. , Devercelli, A. E. , & Devercelli, A. E. (2017). Early childhood development coming of age: Science through the life course. Lancet, 389, 77–90. 10.1016/S0140-6736(16)31389-7 27717614 PMC5884058

[mcn13232-bib-0009] Britto, P. R. , Lye, S. J. , Proulx, K. , Yousafzai, A. K. , Matthews, S. G. , Vaivada, T. , Perez‐Escamilla, R. , Rao, N. , Ip, P. , Fernald, L. C. , & MacMillan, H. (2017). Nurturing care: Promoting early childhood development. Lancet, 389, 91–102. 10.1016/S0140-6736(16)31390-3 27717615

[mcn13232-bib-0010] Britto, P. R. , Yoshikawa, H. , van Ravens, J. , Ponguta, L. A. , Reyes, M. , Oh, S. , Dimaya, R. , Nieto, A. M. , & Seder, R. (2014). Strengthening systems for integrated early childhood development services: A cross‐national analysis of governance. Annals of the New York Academy of Sciences, 1308, 245–255. 10.1111/nyas.12365 24571220

[mcn13232-bib-0011] Buccini, G. , Pedroso, J. , Coelho, S. E. , Ferreira de Castro, G. , Bertoldo, J. , Sironi, A. , Gondim, J. , Venancio, S. I. , Pérez‐Escamilla, R. , Barreto, M. E. , & Gubert, M. B. (2021). Nurturing care indicators for the Brazilian Early Childhood Friendly Municipal Index (IMAPI). Maternal & Child Nutrition, e13155. 10.1111/mcn.13155 33945222 PMC8968942

[mcn13232-bib-0012] Buccini, G. , Venancio, S. I. , & Pérez‐Escamilla, R. (2021). Scaling up of Brazil's Criança Feliz program: An implementation science analysis [published online ahead of print March 18, 2021]. Annals of the New York Academy of Sciences, 1–7. 10.1111/nyas.14589 PMC8349773 33738809

[mcn13232-bib-0013] Câmara dos Deputados . (2016). Avanços do marco legal da primeira infância. Centro de Estudos e Debates Estratégicos, Brasilia‐DF.

[mcn13232-bib-0014] Chirinea, A. M. , & Brandao, C. F. (2015). The IDEB as state regulatory policy and legitimation of quality: In search of meaning. Ensaio: Avaliação e Políticas Públicas em Educação, 23(87), 461–484. 10.1590/S0104-40362015000100019

[mcn13232-bib-0015] Conselho de Desenvolvimento Econômico e Social (CDES) . (2014). As desigualdades na escolarização no Brasil: relatório de observação n° 5. Brasília: Presidência da República. Retrieved from: http://www.cdes.gov.br/Plone/biblioteca/busca/reuniao-plenaria/deliberacao/parecer-de-observacao-5‐as‐desigualdades‐na‐escolarizacao‐no‐brasil‐06‐2014.pdf/view

[mcn13232-bib-0016] Frongillo, E. A. Jr. (1999). Validation of measures of food insecurity and hunger. The Journal of Nutrition, 129(2S Suppl), 506S–509S. 10.1093/jn/129.2.506S 10064319

[mcn13232-bib-0017] Gubert, M. B. , dos Santos, S. M. C. , Santos, L. M. P. , & Pérez‐Escamilla, R. (2017). A municipal‐level analysis of secular trends in severe food insecurity in Brazil between 2004 and 2013. Global Food Security, 14, 61–67.

[mcn13232-bib-0018] Instituto Brasileiro de Geografia e Estatistica (IBGE) . (2017). Living conditions, poverty and inequality. Instituto Brasileiro de Geografia e Estatística. Retrieved from: https://www.ibge.gov.br/en/statistics/multi‐domain/living‐conditions‐poverty‐and‐inequality

[mcn13232-bib-0019] Instituto de Pesquisa Econômica e Aplicada (IPEA) . (2015). In M. A. Costa & B. O. Margu (Eds.), Atlas da vulnerabilidade social nos municípios brasileiros. IPEA.

[mcn13232-bib-0020] Keating, K. , Cole, P. , & Schaffner, M. (2020). The State of Babies Yearbook: 2020. Retrieved from https://stateofbabies.org/wp‐content/uploads/2020/06/State‐of‐Babies‐2020‐Full‐Yearbook‐061820.pdf

[mcn13232-bib-0021] Lin, W. L. , & Yao, G. (2014). Predictive Validity. In A. C. Michalos (Ed.), Encyclopedia of quality of life and well‐being research. Springer. 10.1007/978-94-007-0753-5_2241

[mcn13232-bib-0022] Lu, C. , Black, M. M. , & Richter, L. M. (2016). Risk of poor development in young children in low‐income and middle‐income countries: An estimation and analysis at the global, regional, and country level. The Lancet Global Health, 4, e916–e922. 10.1016/S2214-109X(16)30266-2 27717632 PMC5881401

[mcn13232-bib-0023] Lu, C. , Cuartas, J. , Fink, G. , McCoy, D. , Liu, K. , Li, Z. , Daelmans, B. , & Richter, L. (2020). Inequalities in early childhood care and development in low/middle‐income countries: 2010–2018. BMJ Global Health, 5, e002314. 10.1136/bmjgh-2020-002314 PMC7042597 32133201

[mcn13232-bib-0024] Massardi, W. D. O. , & Abrantes, L. A. (2016). Dependence of municipalities of Minas Gerais in relation to FPM. Gestão e Sociedade, 10(27), 1416–1416.

[mcn13232-bib-0025] Morris, A. S. , Robinson, L. R. , Hays‐Grudo, J. , Claussen, A. H. , Hartwig, S. A. , & Treat, A. E. (2017). Targeting parenting in early childhood: A public health approach to improve outcomes for children living in poverty. Child Development, 88, 388–397. 10.1111/cdev.12743 28138978 PMC5345847

[mcn13232-bib-0026] Mourão, E. , de Oliveira Gallo, C. , do Nascimento, F. A. , & Jaime, P. C. (2019). Temporal trend of Food and Nutrition Surveillance System coverage among children under 5 in the Northern Region of Brazil, 2008‐2017. Epidemiologia e Servicos de Saude: Revista Do Sistema Unico de Saude Do Brasil, 29(2), e2019377. 10.5123/s1679-49742020000200026 32428169

[mcn13232-bib-0027] Neves, J. A. , Vasconcelos, F. , Machado, M. L. , Recine, E. , Garcia, G. S. , & Medeiros, M. (2020). The Brazilian cash transfer program (Bolsa Família): A tool for reducing inequalities and achieving social rights in Brazil [published online ahead of print November 30, 2020]. Global Public Health Advance online publication, 1–17. 10.1080/17441692.2020.1850828 33253042

[mcn13232-bib-0028] Nsoesie, E. O. , Lima Neto, A. S. , Jay, J. , Wang, H. , Zinszer, K. , Saha, S. , Maharana, A. , Marinho, F. , & Soares Filho, A. M. (2020). Mapping disparities in homicide trends across Brazil: 2000–2014. Injury Epidemiology, 7, 47. 10.1186/s40621-020-00273-y 32892747 PMC7487619

[mcn13232-bib-0029] Organization for Economic Co‐operation and Development (OECD) . (2015). Education Policy Outlook Brazil. In: Education Policy Outlook 2015: Making Reforms Happen. Retrieved from: http://www.oecd.org/edu/policyoutlook.htm

[mcn13232-bib-0030] Palmeira, P. A. , Salles‐Costa, R. , & Pérez‐Escamilla, R. (2020). Effects of family income and conditional cash transfers on household food insecurity: evidence from a longitudinal study in Northeast Brazil. Public Health Nutrition, 23(4), 756–767. 10.1017/S1368980019003136 31685079 PMC10200439

[mcn13232-bib-0031] Pérez‐Escamilla, R. , Jimenez, E. Y. , & Dewey, K. G. (2021). Responsive feeding recommendations: Harmonizing integration into dietary guidelines for infants and young children. Current Developments in Nutrition, nzab076. 10.1093/cdn/nzab076 34104850 PMC8178105

[mcn13232-bib-0032] Reis, E. (2014). Spatial income inequality in Brazil, 1872–2000. Economia, 15, 119–140. 10.1016/j.econ.2014.06.006

[mcn13232-bib-0033] Richter, L. M. , Black, M. , Britto, P. , Daelmans, B. , Desmond, C. , Devercelli, A. , Dua, T. , Fink, G. , Heymann, J. , Lombardi, J. , & Lu, C. (2019). Early childhood development: an imperative for action and measurement at scale. BMJ Global Health, 4, e001302. 10.1136/bmjgh-2018-001302 PMC6590994 31297254

[mcn13232-bib-0034] Richter, L. M. , Cappa, C. , Issa, G. , Lu, C. , Petrowski, N. , & Naicker, S. N. (2020). Data for action on early childhood development. The Lancet, 396, 1784–1786. 10.1016/S0140-6736(20)32482-X 33220852

[mcn13232-bib-0035] Richter, L. M. , Daelmans, B. , Lombardi, J. , Heymann, J. , Boo, F. L. , Behrman, J. R. , Lu, C. , Lucas, J. E. , Perez‐Escamilla, R. , Dua, T. , Bhutta, Z. A. , & Paper 3 Working Group and the Lancet Early Childhood Development Series Steering Committee . (2017). Investing in the foundation of sustainable development: pathways to scale up for early childhood development. The Lancet, 389, 103–118. 10.1016/S0140-6736(16)31698-1 PMC5880532 27717610

[mcn13232-bib-0036] Salata, A. (2020). Race, class and income inequality in Brazil: A social trajectory analysis. Dados, 63. 10.1590/dados.2020.63.3.213

[mcn13232-bib-0037] Schober, P. , Boer, C. , & Schwarte, L. A. (2018). Correlation coefficients: Appropriate use and interpretation. Anesthesia & Analgesia, 126(5), 1763–1768. 10.1213/ANE.0000000000002864 29481436

[mcn13232-bib-0038] Sengupta, U. (2019). State‐led housing development in Brazil and India: A machinery for enabling strategy? International Journal of Housing Policy, 19, 509–535. 10.1080/19491247.2018.1510076

[mcn13232-bib-0039] Shonkoff, J. P. , Garner, A. S. , Siegel, B. S. , Dobbins, M. I. , Earls, M. F. , McGuinn, L. , Pascoe, J. , Wood, D. L. , & Committee on Psychosocial Aspects of Child and Family Health, Committee on Early Childhood, Adoption, and Dependent Care . (2012). The lifelong effects of early childhood adversity and toxic stress. Pediatrics, 129, e232–e246. 10.1542/peds.2011-2663 22201156

[mcn13232-bib-0040] Silva, E. S. , & Paes, N. A. (2019). Bolsa Família Programme and the reduction of child mortality in the municipalities of the Brazilian semiarid region. Ciência & Saúde Coletiva, 24, 623–630. 10.1590/1413-81232018242.04782017 30726394

[mcn13232-bib-0041] Silva, J. V. , & Roncalli, A. G. (2020). Trend of social iniquities in reports of sexual violence in Brazil between 2010 and 2014. Revista Brasileira de Epidemiologia, 23, e200038. Epub June 01, 2020. 10.1590/1980-549720200038 32491050

[mcn13232-bib-0042] UNICEF . (2020). Country profiles for early childhood development. Retrieved from: https://nurturing‐care.org/resources/country‐profiles/

[mcn13232-bib-0043] Walker, S. P. , Wachs, T. D. , Grantham‐McGregor, S. , Black, M. M. , Nelson, C. A. , Huffman, S. L. , Baker‐Henningham, H. , Chang, S. M. , Hamadani, J. D. , Lozoff, B. , & Gardner, J. M. (2011). Inequality in early childhood: risk and protective factors for early child development. The Lancet, 378, 1325–1338. 10.1016/S0140-6736(11)60555-2 21944375

[mcn13232-bib-0044] Wehrmeister, F. C. , da Silva, I. C. M. , Barros, A. J. , & Victora, C. G. (2017). Is governance, gross domestic product, inequality, population size or country surface area associated with coverage and equity of health interventions? Ecological analyses of cross‐sectional surveys from 80 countries. BMJ Global Health, 2(4), e000437. 10.1136/bmjgh-2017-000437 PMC5717925 29225951

[mcn13232-bib-0045] World Health Organization . (2019) Operationalizing Nurturing Care for Early Childhood Development: The role of the health sector alongside other sectors and actors. World Health Organization, Geneva: Switzerland.

[mcn13232-bib-0046] World Health Organization (WHO) , UNICEF , & World Bank . (2018). Nurturing Care for Early Childhood Development: A framework for helping children survive and thrive to transform health and human potential. World Health Organization: Geneva. Licence: CC BY‐NC‐SA 3.0 IGO.

