# Supplementary figures and images for: Sociodemographic inequities in nurturing care for early childhood development across Brazilian municipalities
Source: Matern Child Nutr. 2021 Jul 6;18(Suppl 2):e13232. doi: 10.1111/mcn.13232 (PMC8968940; doi:10.1111/mcn.13232)

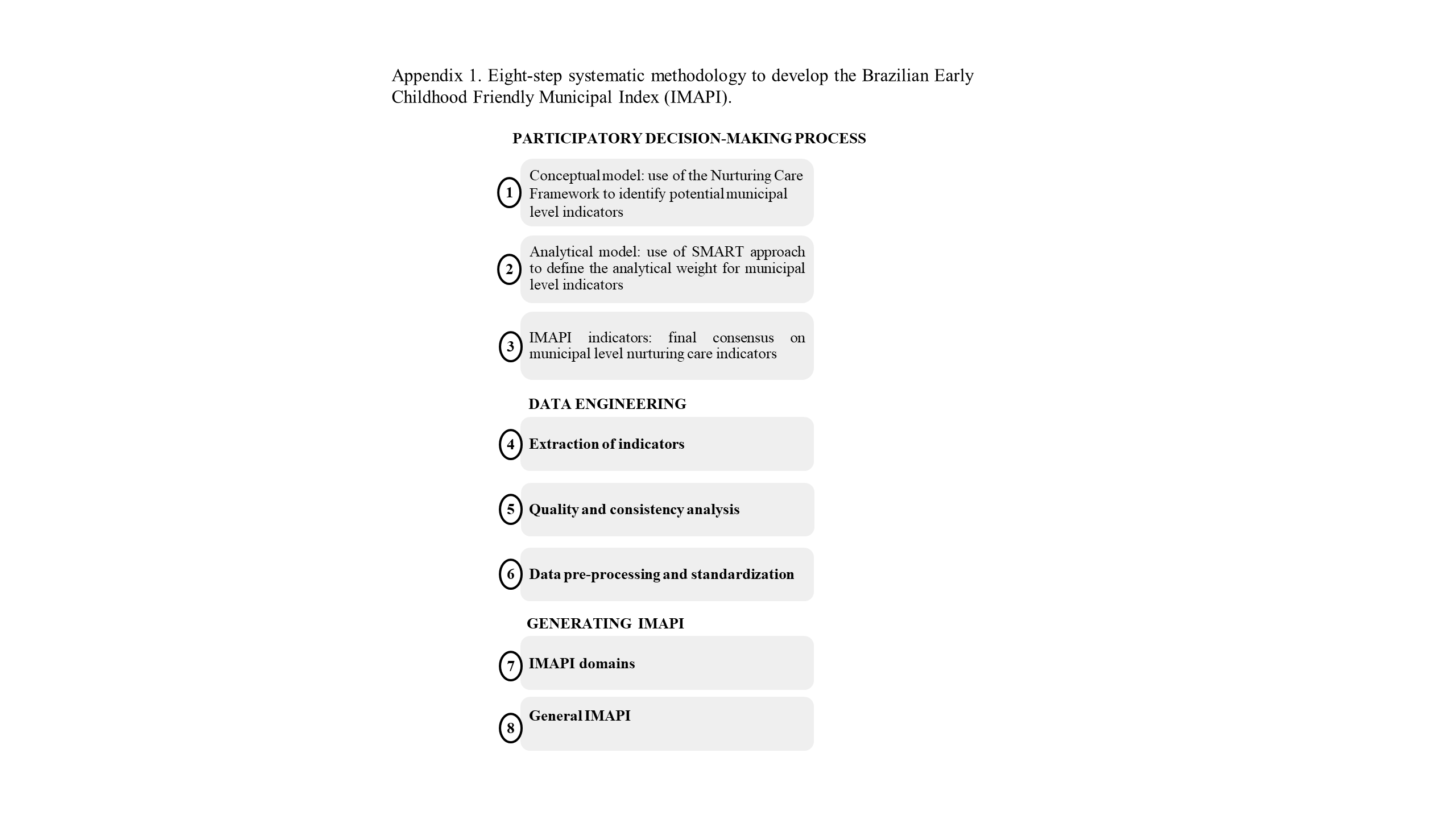

Supplement: Supplementary file 1 — Appendix S1.. Eight‐step systematic methodology to develop the Brazilian Early Childhood Friendly Municipal Index (IMAPI). [file MCN-18-e13232-s003.tif]
