# Supplementary material for: Sociodemographic inequities in nurturing care for early childhood development across Brazilian municipalities
Source: Matern Child Nutr. 2021 Jul 6;18(Suppl 2):e13232. doi: 10.1111/mcn.13232 (PMC8968940; doi:10.1111/mcn.13232)
Supplement: Supplementary file 2 — Appendix S2.. Standard definitions of the selected indicators composing the Brazilian Early Childhood Friendly Municipal Index (IMAPI). [file MCN-18-e13232-s002.docx]

| **Appendix 2.** Standard definitions of the selected indicators composing the Brazilian Early Childhood Friendly Municipal Index (IMAPI). | | | | | | |  |
| --- | --- | --- | --- | --- | --- | --- | --- |
| **Name of the indicator** | **Technical**  **definition** | **Theoretical justification and influence on ECD** | **Calculation method** | **Interpretation** | **Source of information** | **Year of reference** | |
| Prenatal care consultations | Number of pregnant women with 6 or more prenatal care consultations in relation to the total number of pregnant women monitored (number of live births), by year and municipality of residence. | The prenatal period is a sensitive period in the baby’s formation and development. Proper prenatal care, prevention, and early diagnosis of complications can affect child’s development, as well as promote and protect the health of the mother and baby. | (Number of women with 6 or more prenatal care consultations / total live births) * 100, per year and municipality of residence. | An adequate number of prenatal care consultations can protect the child from suboptimal early child development | Sistema de Informação sobre Nascidos Vivos (SINASC/ (Ministry of Health) | 2016 | |
| Early start of prenatal care | Total pregnant women who started prenatal care in the first 12 weeks of pregnancy in relation to the total number of pregnant women monitored, by year and municipality of residence. | The early start of prenatal care facilitates screening for risk factors and the possibility of treating possible complications early, promoting health and adequate fetal development. | (Total pregnant women who started prenatal care at 12 weeks or less / total pregnant women monitored) * 100, per year and municipality of residence | The early start of prenatal care can protect the child from suboptimal early child development | Sistema de Informação sobre Nascidos Vivos (SINASC/ (Ministry of Health) | 2016 | |
| Congenital syphilis | Number of confirmed and notified cases of congenital syphilis in children under 5 years old in relation to the total number of children under 5 years old, by year and municipality of residence. | Congenital syphilis can cause severe changes in the child’s development, including pseudo paralysis of the limbs, neurological deafness, and learning difficulties. | (Number of confirmed and notified cases of congenital syphilis in children under 5 years old / population under 5 years old) * 10,000, per year and municipality of residence. | Congenital syphilis is a risk factor for suboptimal early child development | Numerator: Sistema Nacional de Agravos de Notificação (SINAN / Ministry of Health); and Denominator: Instituto Brasileiro de Geografia e estatística (IBGE). | 2015 | |
| C-Section | Number of births via C-Section in relation to the total number of births, by year and municipality of residence. | High rates of elective C-Section are associated with prematurity and increased maternal and child morbimortality. | (Total live births via C-Section / total live births) * 100, per year and municipality of residence. | C-Section is a risk factor for suboptimal early child development | Sistema de Informação sobre Nascidos Vivos (SINASC/ (Ministry of Health) | 2016 | |
| Home visits in the first 10 days of child’s life | Percentage of primary care teams in the municipality that participated in the second cycle of the PMAQ (National Program for Improving Access and Quality in Primary Care) and reported carrying out home visits in the first 10 days of child’s life. | Home visits by the health team in the first ten postpartum days help to detect, prevent, and quickly treat postpartum and breastfeeding complications (adequate nutrition). Home visits by health teams also reflect the mother’s and newborn’s access and ties with the health service. | (Number of teams that conduct Home visits in the first 10 days of child’s life / total teams that participated in the PMAQ 2nd cycle) * 100, per year and municipality of residence. | Home visits in the first 10 days of child’s life can protect the child from suboptimal early child development | Programa Nacional de Melhoria do Acesso e da Qualidade da Atenção Básica (PMAQ 2º Ciclo) /  Ministry of Health | 2013 / 2014 | |
| Child hospitalization for pneumonia or gastroenteritis | Percentage of children under 5 years old hospitalized for pneumonia or gastroenteritis in relation to the total number of children under 5 years old, by year and municipality of residence. | Gastroenteritis and acute respiratory diseases result from a set of biological, environmental, and socio-cultural variables and indicate a high degree of vulnerability, which can cause greater risks and compromises child development. These are conditions that could be avoided or reduced by effective primary care (prevention, diagnosis, and early treatment). | Total children under 5 years old hospitalized for pneumonia or gastroenteritis / total children under 5 years old) * 100, per year and municipality of residence. | Child hospitalization for pneumonia or gastroenteritis is a risk factor for suboptimal early child development | Numerator: Sistema de Informações Hospitalares (SIH/Ministry of Health) and Denominator: Instituto Brasileiro de Geografia e Estatística (IBGE). | 2015 | |
| Adolescent pregnancy | Prevalence of pregnant adolescents (10 to 19 years), by year and municipality of residence. | Adolescent pregnancy is a risky situation for the health of the adolescent and the newborn, with an increased chance of intrauterine growth retardation and developmental delays. | (Number of live births to mothers aged 10 to 19 years old / total live births) * 100, per year and municipality of residence. | Adolescent pregnancy is a risk factor for suboptimal early child development | Sistema de Informação sobre Nascidos Vivos (SINASC/Ministry of Health) | 2016 | |
| Low birth weight | Percentage of live births weighing less than 2500 g in relation to the total number of births, by year and municipality of residence. | Low birth weight is a risk factor for inadequate child development, generating negative effects for mental and motor development as well as growth. It is also an indicator of poor prenatal care, maternal malnutrition, adolescent pregnancy, and untreated infections. | (Total live births weighing less than 2500 g / total live births) * 100, per year and municipality of residence. | Low birth weight is a risk factor for suboptimal early child development | Sistema de Informação sobre Nascidos Vivos (SINASC/Ministry of Health). | 2016 | |
| Child mortality | Number of deaths of children under 5 years old for every 1000 live births, per year and municipality of residence. | Most childhood deaths are concentrated in the first year of life, with a high share of perinatal causes (factors linked to pregnancy, childbirth and postpartum), which are generally preventable. After the first year of life, mortality is associated with exposure to environmental factors, for example lack of access to quality health care and other environmental stressors related to poverty. This indicator also expresses the failure in several public policies to promote healthy and complete child development. | (Number of deaths of children under 5 years old / total live births) * 1,000, per year and municipality of residence. | Higher child mortality rates are associated with negative exposure to environmental factors, which are risk factors for suboptimal early child development | Numerator: Sistema de Informação sobre Mortalidade (SIM/ Ministry of Health) and Denominator: Sistema de Informação sobre Nascidos Vivos (SINASC/Ministry of Health). | 2016 | |
| Preventable deaths in children under one year old | Deaths that could be prevented by the performance of health care services in children under one year old in relation to the total number of live births, by year and municipality of residence. Deaths caused by the following categories are considered “preventable”: a) Reducible by immunizations; b) Reducible by caring for women during pregnancy; c) Reducible by adequate care for women during childbirth; d) Reducible by actions, diagnosis, and appropriate treatment; e) Reducible by health promotion activities linked to Primary Health Care. | Preventable deaths occur when support services fail to identify early and correctly intervene in a given problem. Thus, a child who dies from a preventable cause is a child who has been deprived of development due to problems involving the health services of a particular municipality. | (Number of preventable deaths in children under 1 year / total live births) * 1000, per year and municipality of residence. | Higher numbers of preventable deaths in children under one year old are a reflect of failures in the support services, which are risk factors for suboptimal early child development | Numerator:  Sistema de Informação sobre Mortalidade (SIM/ Ministry of Health).  Denominator: Sistema de Informação sobre Nascidos Vivos (SINASC/ Ministry of Health). | 2016 | |
| Prematurity | Percentage of children born at less than 37 completed weeks of gestation in relation to the total number of births, by year and municipality of residence. | Premature birth is a risk factor for inadequate child development in the early years as well as an indicator of poor prenatal care. | (Total children born before 37 completed weeks of gestation / total live births) * 100, per year and municipality of residence. | Prematurity is a risk factor for suboptimal early child development | Sistema de Informação sobre Nascidos Vivos (SINASC/Ministry of Health). | 2016 | |
| Maternal mortality | Number of women who died from causes related to pregnancy, childbirth, or puerperium for every 100,000 live births, per year and municipality of residence. | Maternal death can increase the risks of child mortality, morbidity, and developmental delay in early childhood. | (Number women who died due to causes related to pregnancy, childbirth, and puerperium / number of live births) * 100,000, per year and municipality of residence. | Maternal mortality is a risk factor for suboptimal early child development | Numerator: Sistema de Informação sobre Mortalidade (SIM/Ministry of Health) and Denominator: Sistema de Informação sobre Nascidos Vivos (SINASC/Ministry of Health). | 2016 | |
| Coverage of child immunization | Percentage of children who received first dose of DTaP immunization (triple bacterial vaccine), by year and municipality of residence. | Good vaccination coverage guarantees the maintenance of low incidences of immunopreventable illnesses that can influence children’s development. | (number of doses of the first DTaP immunization (triple bacterial vaccine) / target population) * 100, per year and municipality of residence. | Child immunization is associated with low incidences of immune preventable illnesses, protecting the child from suboptimal early child development | Sistema de Informações do Programa Nacional de Imunizações (SI-PNI / Ministry of health) | 2016 | |
| Coverage of Primary Health Care | Percentage of the population covered by Primary Health Care, by year and municipality. | The population coverage by Primary Health Care teams reflects access to health services. Primary Health Care is responsible for several actions to prevent and promote adequate child development and early diagnosis of complications. | [number of Family Health teams * 3,450 + (number of parameterized Primary Care teams + number of equivalent Family Health teams) ∗ 3,000] in the reference month (December) / Population estimate * 100. | Coverage of Primary Health Care can protect the child from suboptimal early child development | Plataforma e-Gestor Atenção Básica/Ministry of Health. | 2016 | |
| Coverage of information on child feeding practices | Percentage of children under 5 years old with at least one record of information on food consumption (breastfeeding, quality, and diversity of the diet) in the Food and Nutrition Surveillance System (SISVAN), by year and municipality of residence. | Adequate food consumption is associated with better child growth and development. The registration of information on food consumption was used as a monitoring proxy. | (Children under 5 years old with at least 1 record on food consumption in SISVAN / population under 5 years old) * 100, per year and municipality of residence. | Coverage of information on child food consumption is a monitoring proxy of an adequate food consumption, which can protect the child from suboptimal early child development | Numerator: Sistema de Vigilância Alimentar e Nutricional (SISVAN/ Ministry of Health) and Denominator: Instituto Brasileiro de Geografia e Estatística (IBGE). | 2015 | |
| Severe Household Food Insecurity | Estimated prevalence of severe food insecurity in a given municipality. Severe food insecurity is characterized by children’s quantitative reduction in food, disruption in eating patterns resulting from lack of food, and hunger caused by the inability to buy food due to the lack of money. | Children exposed to hunger in the home environment are very likely to experience developmental delays. The prediction of severe food insecurity was used as a proxy for hunger in the municipality. | Estimated prevalence of severe food insecurity in the municipality. | Severe Household Food Insecurity is a risk factor for suboptimal early child development | Gubert MB, Pérez-Escamilla R. Severe food insecurity in Brazilian Municipalities, 2013. DOI: 10.1590/1413-812320182310.265120161 | 2013 | |
| Brazilian Breastfeeding and Feeding Strategy ^a^ | The indicator Brazilian Breastfeeding and Feeding Strategy (EAAB) is an indicator composed a workshop and/or training for instructors and/or certification of the basic health unit in the municipality. | The practice of breastfeeding and the introduction of healthy complementary feeding are associated with adequate child development. The Brazilian Breastfeeding and Feeding Strategy aims to qualify the actions to promote breastfeeding and healthy complementary food, improving the skills and abilities of health professionals for these activities, which should be monitored. | [(∑ Workshop held, training instructors, certified health unit in the municipality) / 3] | Brazilian Breastfeeding and Feeding Strategy  qualifies the actions to promote breastfeeding and healthy complementary food, which can protect the child from suboptimal early child development | Coordenação-Geral de Alimentação e Nutrição (CGAN)/Departamento de Atenção Básica (DAB)/ Ministry of Health | *2013-2019 | |
| Coverage of information on child nutritional status | Percentage of children under 5 years of age with at least one record of information on nutritional status (BMI/age) in the Food and Nutrition Surveillance System (SISVAN), by year and municipality of residence. | Adequate nutritional status is associated with better child development and, thus, must be monitored. The coverage of recorded information about nutritional status was used as a monitoring proxy. | (Children under 5 years old with at least 1 record of nutritional status in SISVAN / population under 5 years old) * 100, per year and municipality of residence. | Coverage of information on child nutritional status is a monitoring proxy of an adequate nutritional status, which can protect the child from suboptimal early child development | Numerator: Sistema de Vigilância Alimentar e Nutricional (SISVAN/ Ministry of Health) and Denominator: Instituto Brasileiro de Geografia e Estatística (IBGE). | 2015 | |
| Visits by national home-visiting parenting skills program ^c^ | Percentage of individuals visited by the national home-visiting parenting skills program in the municipality in relation to the target agreed for the year. | These home visits focus on strengthening parenting skills, especially for socially vulnerable parents, help these families better understand the child development process, as well as how to behave and respond to provide adequate development. In addition, they help identify problems and difficulties and, if necessary, seek additional support services. | [(Total number of individuals visited in the municipality in 2019/goal of the municipality in 2019)*100] | Visits by national home-visiting parenting skills program can strengthening parenting skills, which protect the child from suboptimal early child development | Coordenação do Programa Criança Feliz/ Ministry of Citizenship. | 2019 | |
| Coverage of daycare and preschool | Number of enrollments in daycare and preschool in relation to the total number of children under 5 years, by year and municipality of residence. | Children need access to free, quality daycare and preschools, where they will receive, under adequate supervision, the stimuli and care necessary for development. | [(∑ daycare enrollment and preschool enrollment) / (population under 5 years old)] * ​​100, per year and municipality. | Coverage of daycare and preschool can protect the child from suboptimal early child development | Censo Escolar (School Census) | 2015 | |
| Number of students per daycare professional | Number of students enrolled for each professional employed in public and private daycares in the municipality per year. | The number of children per professional should enable all children to receive the attention, responsibility, and interaction necessary for adequate development. | (Number of daycare enrollments / number of daycare professionals) * 100, per year and municipality. | A higher number of students per daycare professional is a risk factor for suboptimal early child development | Censo Escolar (School Census) | 2016 | |
| Number of students per preschool professional | Number of students enrolled for each professional employed in public and private preschools in the municipality per year. | The number of children per professional should enable all children to receive the attention, responsibility, and interaction necessary for adequate development. | (Number of preschool enrollments/number of preschool professionals) * 100, per year and municipality. | A higher number of students per preschool professional is a risk factor for suboptimal early child development | Censo Escolar (School Census) | 2016 | |
| Percentage of qualified daycare teachers | Percentage of teachers with higher education employed in public and private daycares in the municipality per year. | Teachers with specific and qualified training are better prepared to understand the needs and provide early stimulation to children that supports their proper development. | (Number of teachers with higher education in daycares / total number of daycare teachers) * 100, per year and municipality. | A higher percentage of teachers with higher education in daycares can protect the child from suboptimal early child development | Censo Escolar (School Census) | 2016 | |
| Percentage of qualified preschool teachers | Percentage of teachers with higher education employed in public and private preschools in the municipality per year. | Teachers with specific and qualified training are better prepared to understand the needs and provide early stimulation to children that supports their proper development. | (Number of teachers with higher education in preschools / total number of preschool teachers) * 100, per year and municipality. | A higher percentage of teachers with higher education in preschools can protect the child from suboptimal early child development | Censo Escolar (School Census) | 2016 | |
| Daycare educational resources | Presence of library/study room and/or playground and/or children’s restroom in daycares in the municipality per year. | Daycares must have adequate infrastructure for children, such as the presence of a reading room, playgrounds, adapted restrooms, and educational material available. An adequate infrastructure is fundamental for the stimulation and learning of children, thus favoring complete and adequate development. | (∑ Number of library/study rooms, playgrounds, and children’s restrooms in daycare) / total daycare, by year and municipality. | The availability of daycare educational resources can protect the child from suboptimal early child development | Censo Escolar (School Census) | 2016 | |
| Preschools educational resources | Presence of library/study room and/or playground and/or children’s restroom in schools for early childhood in the municipality per year. | Preschools must have adequate infrastructure for children, such as the presence of a reading room, playgrounds, adapted restrooms, and educational material available. An adequate infrastructure is fundamental for the stimulation and learning of children, thus favoring complete and adequate development. | (∑ Number of library/study rooms, playgrounds, and children’s restrooms in preschools) / total number of preschools, by year and municipality | The availability of preschool educational resources can protect the child from suboptimal early child development | Censo Escolar (School Census) | 2016 | |
| Coverage of the National conditional cash transfer program ^b^ | Percentage of families benefiting from the National conditional cash transfer program among families in the Brazilian single registry with children under 5 years old. | Cash transfer programs protect the development of children in extreme poverty. The coverage of the National conditional cash transfer program reflects the percentage of children in situations of poverty assisted in a social program. | (Total families benefiting from National conditional cash transfer program with children under 5 years old in the reference month (December) / total families enrolled in the Brazilian single registry with children under 5 years old, in the reference month (December)) * 100, per municipality. | The access to the National conditional cash transfer program can protect the child from suboptimal early child development | Cadastro Único (CadÚnico/ Ministry of Citizenship). | 2016 | |
| Air pollution | Estimated daily concentration of fine particulate matter (PM2.5) (ug/m3) by municipality. | During pregnancy and early childhood, the rapid development of children makes them especially vulnerable to environmental stressors. Exposure to air pollution is a stressor that can affect children of all social classes, facilitating the development of childhood respiratory diseases, which can impact cognitive development and increase susceptibility to diseases in general. | Estimation of the daily concentration of fine particulate matter (PM2.5) (ug/m3) from socio-temporal models by municipality. | Air pollution is a risk factor for suboptimal early child development | Brentani A et al. Child Development Center. Faculty of Medicine. University of Sao Paulo. | 2015 | |
| Notification of violence against women | Total reported cases of any type of violence against women of childbearing age (10 to 49 years) in relation to the number of women in this age group in the municipality per year. | Violence against women can result in several health problems such as abortion, low birth weight, and prematurity. The notification of violence by the health service is a method to create a qualified service network against chronic exposure to violence, which impacts child development. | (Number of cases with ICD Y09 (aggression by unspecified means) in women between 10 and 49 years old / population of women between 10 and 49 years old) * 1000, per year and municipality. | The notification of violence against women create a qualified service network against chronic exposure to violence, which can protect the child from suboptimal early child development | Numerator: Sistema Nacional de Agravos de Notificação (SINAN/ Ministry of Health) and Denominator: Instituto Brasileiro de Geografia e Estatística (IBGE). | 2015 | |
| Notification of violence against children | Total reported cases of any type of violence against children under 5 years old in relation to the total number of children under 5 in the municipality per year. | The notification of violence by the health service is a method to create a qualified service network against the child’s chronic exposure to violence, which impacts child development. | (Number of cases with ICD Y09 (aggression by unspecified means) for individuals under 5 years old / population under 5 years old) * 1000, per year and municipality. | The notification of violence against children create a qualified service network against chronic exposure to violence, which can protect the child from suboptimal early child development | Numerator: Sistema Nacional de Agravos de Notificação (SINAN/ Ministry of Health) and Denominator: Instituto Brasileiro de Geografia e Estatística (IBGE). | 2015 | |
| Homicides | Homicide rate estimated per 100 thousand inhabitants for each municipality. | Early exposure to violent environments can impair childhood development, including brain development, and damage other parts of the nervous system with lifelong consequences. A safe environment with low levels of violence allows the children and their caregivers to be safe and protected, free to come and go, which permits them to explore the environment around them. | (∑ Number of deaths due to aggression, number of deaths caused by legal intervention, number of hidden homicides / municipal population) * 100,000, per year and municipality. | A higher homicide rate is a risk factor for suboptimal early child development | Instituto de Pesquisa Econômica Aplicada (IPEA - Institute of Applied Economic Research) - Atlas da Violência (Atlas of Violence). | 2017 | |
| Portuguese name of programs: ^a^ *Estratégia Amamenta e Alimenta Brasil;* ^b^ *Programa Bolsa Família;* ^c^ *Programa Criança Feliz*. Color-code: Good Health domain/yellow, Adequate Nutrition domain/pink, Responsive Care domain/green, Opportunities for Early Learning domain/red, Safety and Security domain/blue. | | | | | | |  |
