# Supplementary material for: Sociodemographic inequities in nurturing care for early childhood development across Brazilian municipalities
Source: Matern Child Nutr. 2021 Jul 6;18(Suppl 2):e13232. doi: 10.1111/mcn.13232 (PMC8968940; doi:10.1111/mcn.13232)
Supplement: Supplementary file 3 — Appendix S3.. Technical note on methods used to impute, standardize, and calculate IMAPI scores [file MCN-18-e13232-s001.docx]

**Appendix 3.** **Technical note on methods used to impute, standardize, and calculate IMAPI scores**

This supplementary material briefly describes the imputation and standardization methods applied to the nurturing care indicators selected to compose IMAPI scores and the final computation of the overall IMAPI score and five sub-scores, representing one of the five Nurturing Care Framework domains.

1. **Imputation**

Nine out of the 31 nurturing care indicators selected in the IMAPI had missing information as presented in Box 1. The missingness mechanism for each indicator was checked to better decide how to address missing data. Missing data were determined to be completely at random, meaning that there is no relationship between the missing value of a given data and the values of the other data. Thus, we used a multiple-data imputation strategy using the Multivariate Imputation by Chained Equations (MICE method) as suggested in the literature (Van Buuren et al., 1999; Azur et al., 2011). After data imputation, density plots were used to assess the similarity of the new distributions of observed and imputed data, which resulted in an accurate construction of all the indicators defined for each domain.

| **Box 1.** Nurturing care indicators with missing information | | |
| --- | --- | --- |
| **Nurturing Care Domain** | **Indicator** | **Number (%) of municipalities with missing information** |
| **Good Health** | Child immunization | 15 (2.69) |
|  | Home visits in the first 10 days of a child’s life | 528 (9.47) |
| **Adequate Nutrition** | Severe Household Food Insecurity | 5 (0.09) |
| **Early Learning** | Percentage of qualified daycare teachers | 415 (7.45) |
|  | Number of students per daycare professional | 341 (6.12) |
|  | Percentage of educational resources in daycares | 479 (8.89) |
|  | Percentage of educational resources in preschools | 14 (0.25) |
| **Security and Safety** | National conditional cash transfer program | 2 (0.03) |
|  | Air pollution | 4 (0.07) |

1. **Standardization**

Two types of standardizations were used to harmonize nurturing care indicators before estimating IMAPI scores: (1) Inversion of attributes: indicators representing risk factors for early childhood development (i.e., the higher the values of these indicators, the worse the ECD would be) were reversed by multiplying them by -1 to harmonize their interpretation within IMAPI (see Box 2); (2) Standardization of outliers: for indicators expressed in percentage, we considered any values above 100% as outliers and applied a rule of standardizing it to 100%. For example, a municipality with child immunization of 120% was rounded to 100%.

| **Box 2.**  Nurturing care indicators multiplied by -1 because they were considered a risk factor due to their negative impact on Early Childhood Development. | |
| --- | --- |
| **Nurturing Care Domain** | **Indicator** |
| **Good Health** | Congenital syphilis |
|  | Child mortality |
|  | C-Section |
|  | Child hospitalization for pneumonia or gastroenteritis |
|  | Preventable deaths in children under one-year-old |
|  | Adolescent pregnancy |
|  | Prematurity |
|  | Low birth weight |
|  | Maternal mortality |
| **Adequate Nutrition** | Severe Household Food Insecurity |
| **Opportunities for early learning** | Number of students per daycare professional |
|  | Number of students per preschool professional |
| **Security and Safety** | Air Pollution |
|  | Homicides |

1. **IMAPI scores calculation**

C.1. IMAPI domains. Considering the statistical criteria for at least two indicators to compute IMAPI domain scores, the Responsive Caregiving domain was not included in the overall IMAPI score.

Domains scores (DS) were calculated using the weighted arithmetic mean of standardized values of indicators (indicated as I_n_) and the indicator analytical weight (I*_n-weight_)*^[[1]](#footnote-1)^, as follows: IMAPI DS = (I_1_*I_1-_*_weight_*)+ (I_2_*I_2-_*_weight_*)+… (I_n_*I_n-_*_weight_*)/ I_1-_*_weight_* + I_2-_*_weight_* +… I_n-_*_weight._* Final values of each domain were normalized between 0 and 100 through rescaling (min-max normalization) (Han 2011), and scores were categorized as high, medium, and low based on the corresponding tercile distribution (see Box 3).

C.2. Overall IMAPI. Following the calculation of each domain score (DS*_n_*), the overall IMAPI score was calculated based on the average of the DS, as follows: overall IMAPI= (DS_good health_)+ (DS_adequate nutrition_)+( DS_early learning_)+(DS_security and safety_)/4*_._* The overall IMAPI score ranged from 0 to 100, and scores were categorized in high, medium, and low categories based on the corresponding tercile distributions (see Box 3).

| Box 3. Distribution of municipalities within overall IMAPI score and domain sub-scores. | | | | | | |
| --- | --- | --- | --- | --- | --- | --- |
| **IMAPI score and domain sub-scores** | Tercile cut-off points | | | **Number of municipalities**  *% (n)* | | |
|  | **Low** | **Medium** | **High** | *Low* | *Medium* | *High* |
| Overall IMAPI | 0–38 | 39–44 | 45–100 | 39.0 (2170) | 29.8 (1658) | 31.3 (1742) |
| Good health | 0–54 | 55–63 | 64–100 | 34.7 (1935) | 32.9 (1830) | 32.4 (1805) |
| Adequate nutrition | 0–22 | 23–28 | 29–100 | 38.2 (2129) | 29.8 (1658) | 32.0 (1783) |
| Opportunities for early learning | 0–62 | 63–75 | 76–100 | 33.6 (1872) | 34.3 (1911) | 32.1 (1787) |
| Security and safety | 0–20 | 21–25 | 26–100 | 38.0 (2119) | 35.4 (1970) | 26.6 (1481) |
| Responsive Caregiving* | 0–0 | 1–58 | 59–100 | 54.3 (3025) | 12.7 (705) | 33.0 (1840) |
| * not included in the calculation of IMAPI | | | |  |  |  |

**References:**

Azur, M.J., Stuart, E.A., Frangakis, C. and Leaf, P.J. (2011).Multiple imputations by chained equations: what is it, and how does it work? *Int. J. Methods Psychiatr*. Res., 20: 40-49.  <https://doi.org/10.1002/mpr.329>

van Buuren, S., Boshuizen, H. C., & Knook, D. L. (1999). Multiple imputations of missing blood pressure covariates in survival analysis. *Statistics in medicine*, 18(6), 681–694.

Han, J., Pei, J., Kamber, M. (2011). *Data mining: concepts and techniques.* The Morgan Kaufmann Series in Data Management Systems, Elsevier. <https://books.google.com.br/books?id=pQws07tdpjoC>

Buccini G., Pedroso J., Coelho S.E., Ferreira de Castro G., Bertoldo J., Sironi A., et al. (2021b) Nurturing care indicators for the Brazilian Early Childhood Friendly Municipal Index (IMAPI). *Matern Child Nutr.* <http://doi.org/10.1111/mcn.13155>

1. Indicator analytical weight (I*_n-weight_*) corresponds to a consultation with a group of experts through a SMART approach to qualify quality performance of each indicator and can range from 1 (low) to 5 (high) quality performance. Methodological aspects were described in detailed in Buccini et al. 2021b [↑](#footnote-ref-1)
