# Supplementary material for: Sociodemographic inequities in nurturing care for early childhood development across Brazilian municipalities
Source: Matern Child Nutr. 2021 Jul 6;18(Suppl 2):e13232. doi: 10.1111/mcn.13232 (PMC8968940; doi:10.1111/mcn.13232)
Supplement: Supplementary file 4 — Appendix S4.. Demographic characteristics of the 5.570 Brazilian municipalities included in the IMAPI. [file MCN-18-e13232-s004.docx]

| Appendix 4. Demographic characteristics of the 5.570 Brazilian municipalities included in IMAPI. | | |
| --- | --- | --- |
| **Characteristics of the municipalities** | **n** | **%** |
| **Regions** | | |
| North | 450 | 8.1 |
| Northeast | 1794 | 32.2 |
| Southeast | 1668 | 29.9 |
| South | 1191 | 21.4 |
| Central-West | 467 | 8.4 |
| **Population Size** |  |  |
| Very small | 3811 | 68.4 |
| Small | 1100 | 19.7 |
| Medium | 350 | 6.3 |
| Large | 292 | 5.2 |
| Metropolis | 17 | 0.3 |
| **Proportion (%) of vulnerable children** | *mean* | 51.6 |
| Very Low | 727 | 13.1 |
| Low | 741 | 13.3 |
| Medium | 751 | 13.5 |
| High | 718 | 12.9 |
| Very High | 2630 | 47.2 |
|  | | |
